# Supplementary figures and images for: A remodeled RNA polymerase II complex catalyzing viroid RNA-templated transcription
Source: PLoS Pathog. 2022 Sep 19;18(9):e1010850. doi: 10.1371/journal.ppat.1010850 (PMC9521916; doi:10.1371/journal.ppat.1010850)

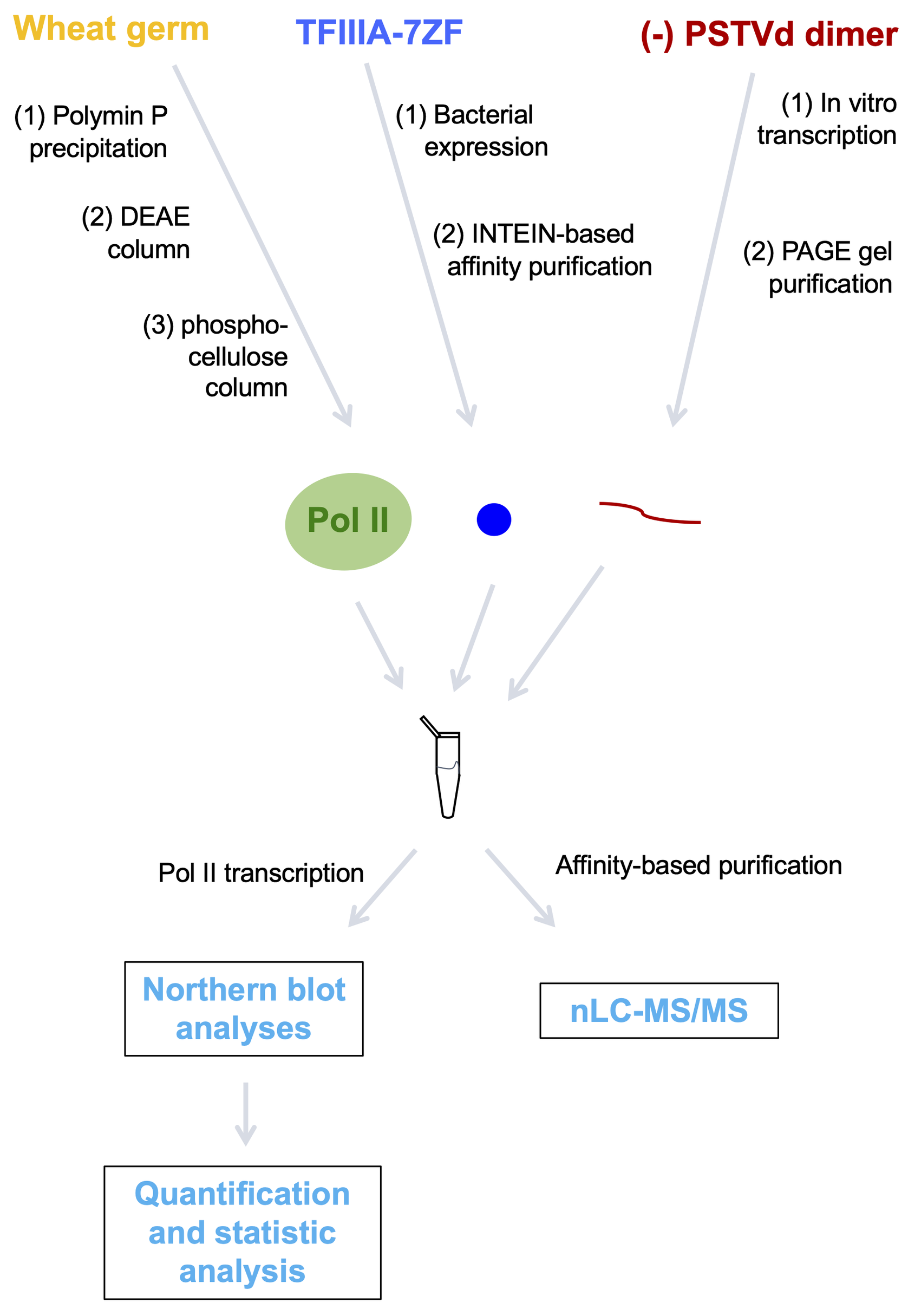

Supplement: S1 Fig — (TIF) [file ppat.1010850.s003.tif]

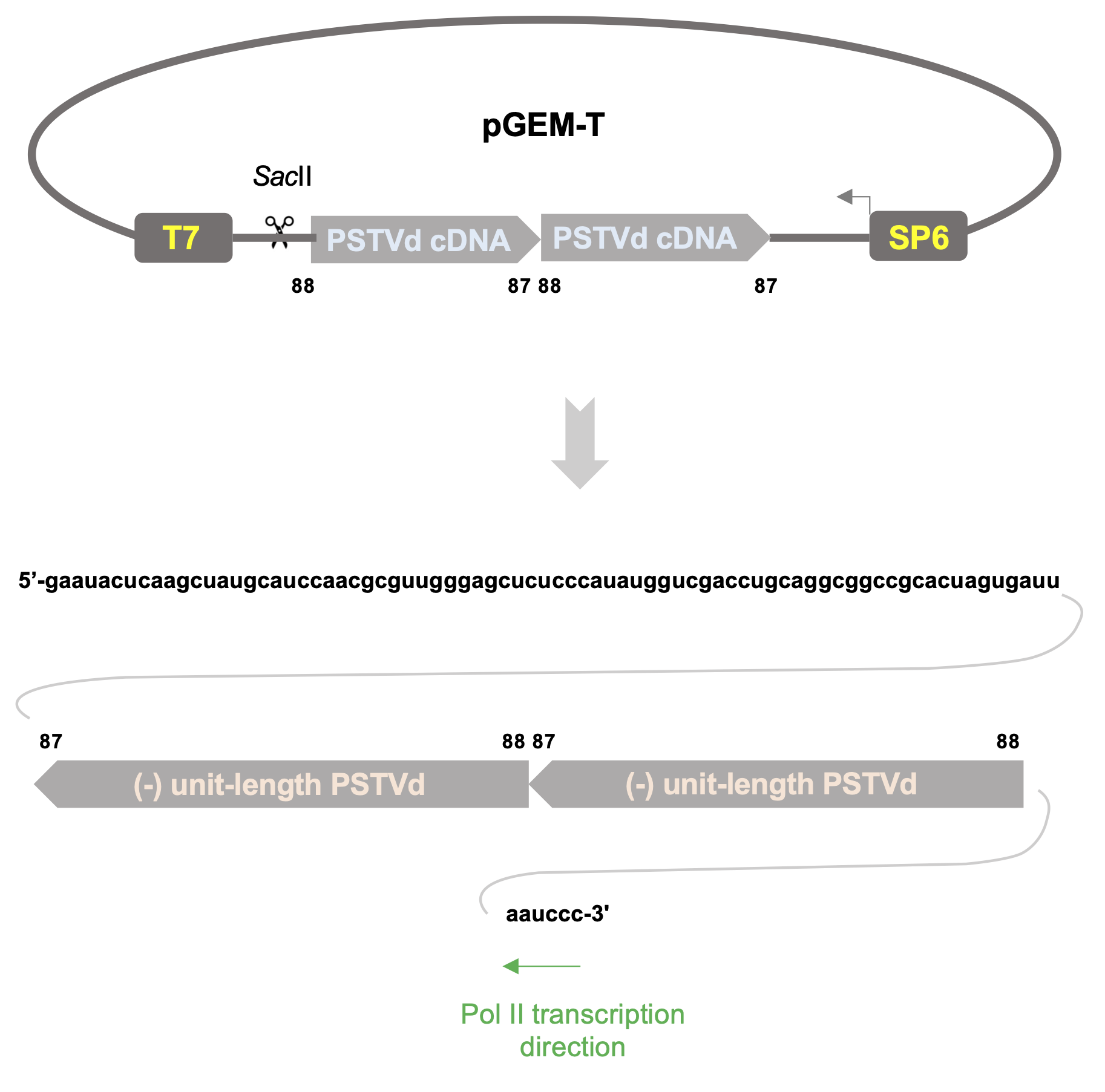

Supplement: S2 Fig — (TIF) [file ppat.1010850.s004.tif]

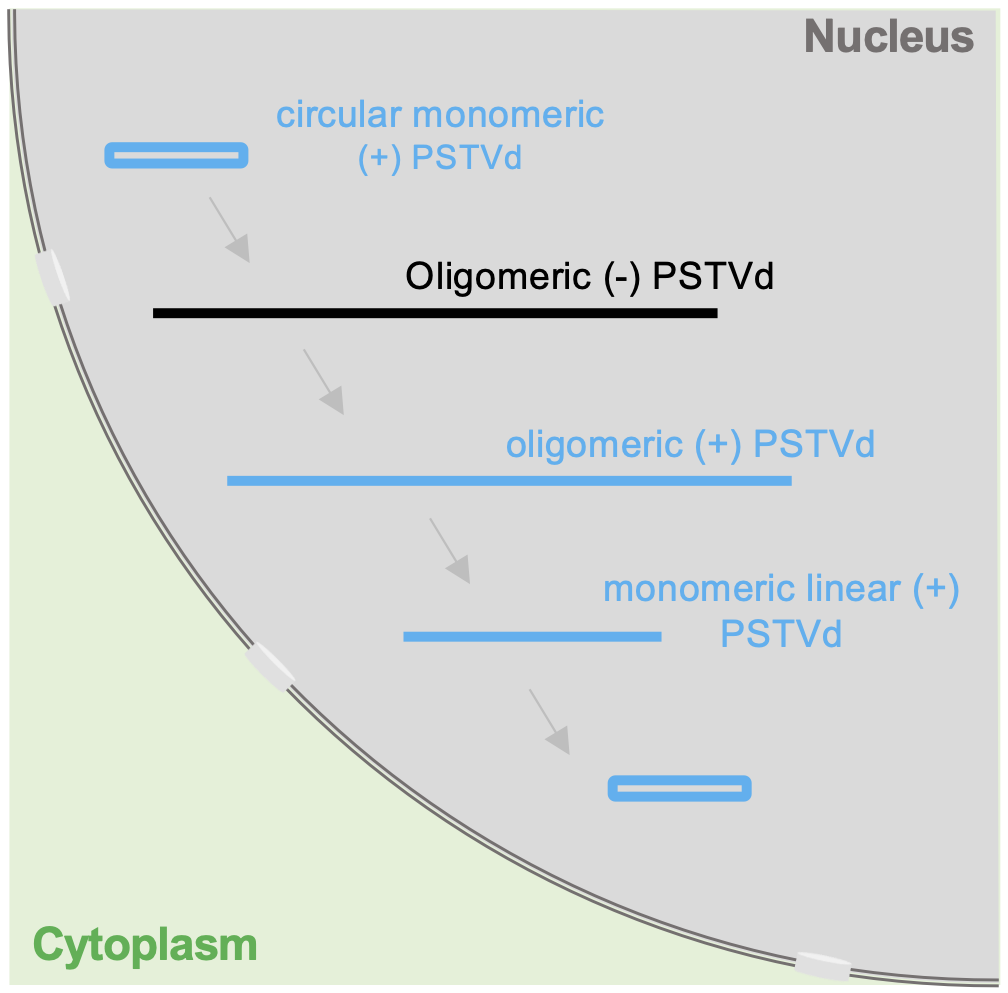

Supplement: S3 Fig — (TIF) [file ppat.1010850.s005.tif]

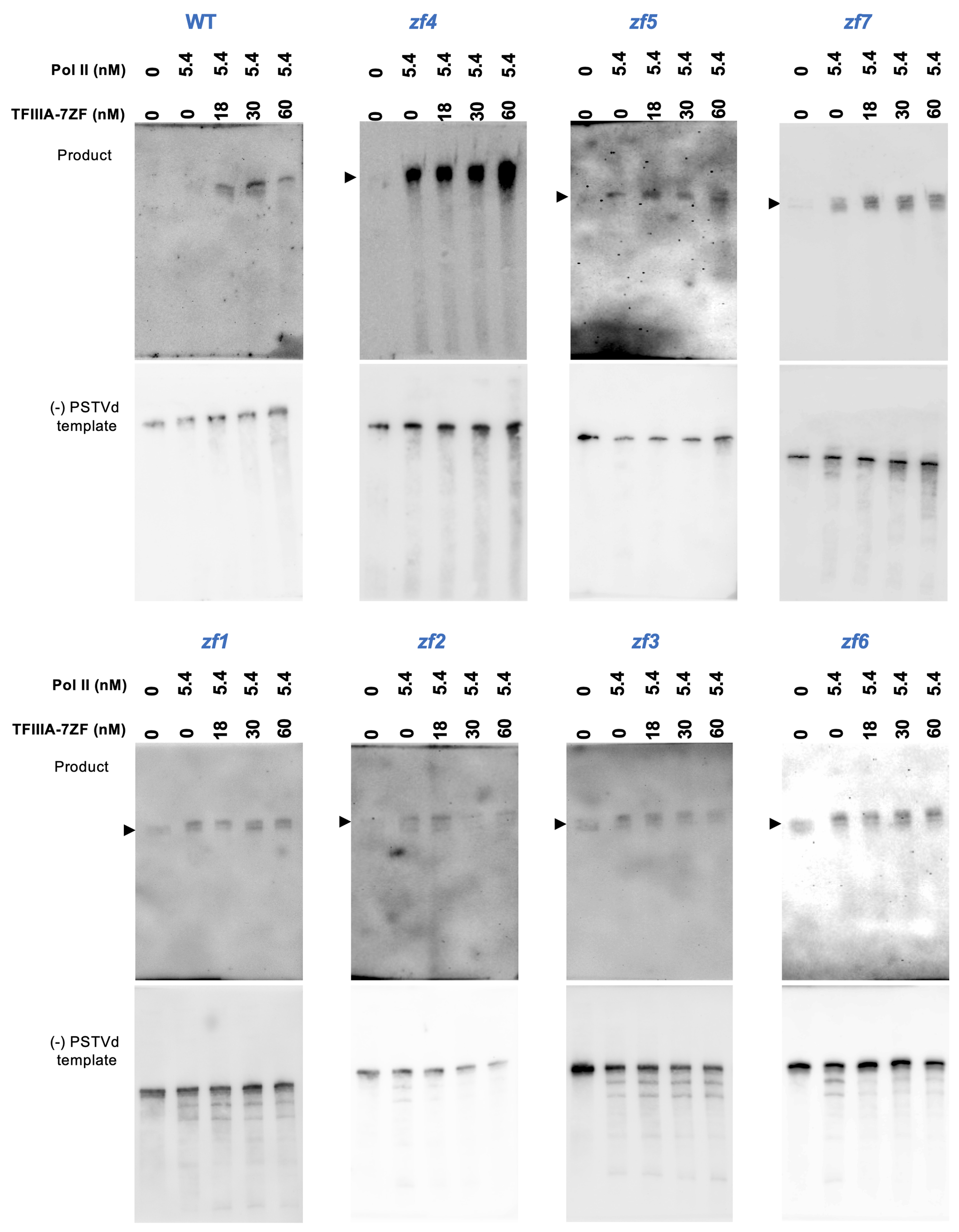

Supplement: S4 Fig — Arrowheads indicate the position of (-) PSTVd dimer template, based on the extremely low cross-reaction signals that were only visible after over-exposure. The product signals were often retarded as compared with the template, likely due to the high-salt/high-volume loading effect. (TIF) [file ppat.1010850.s006.tif]

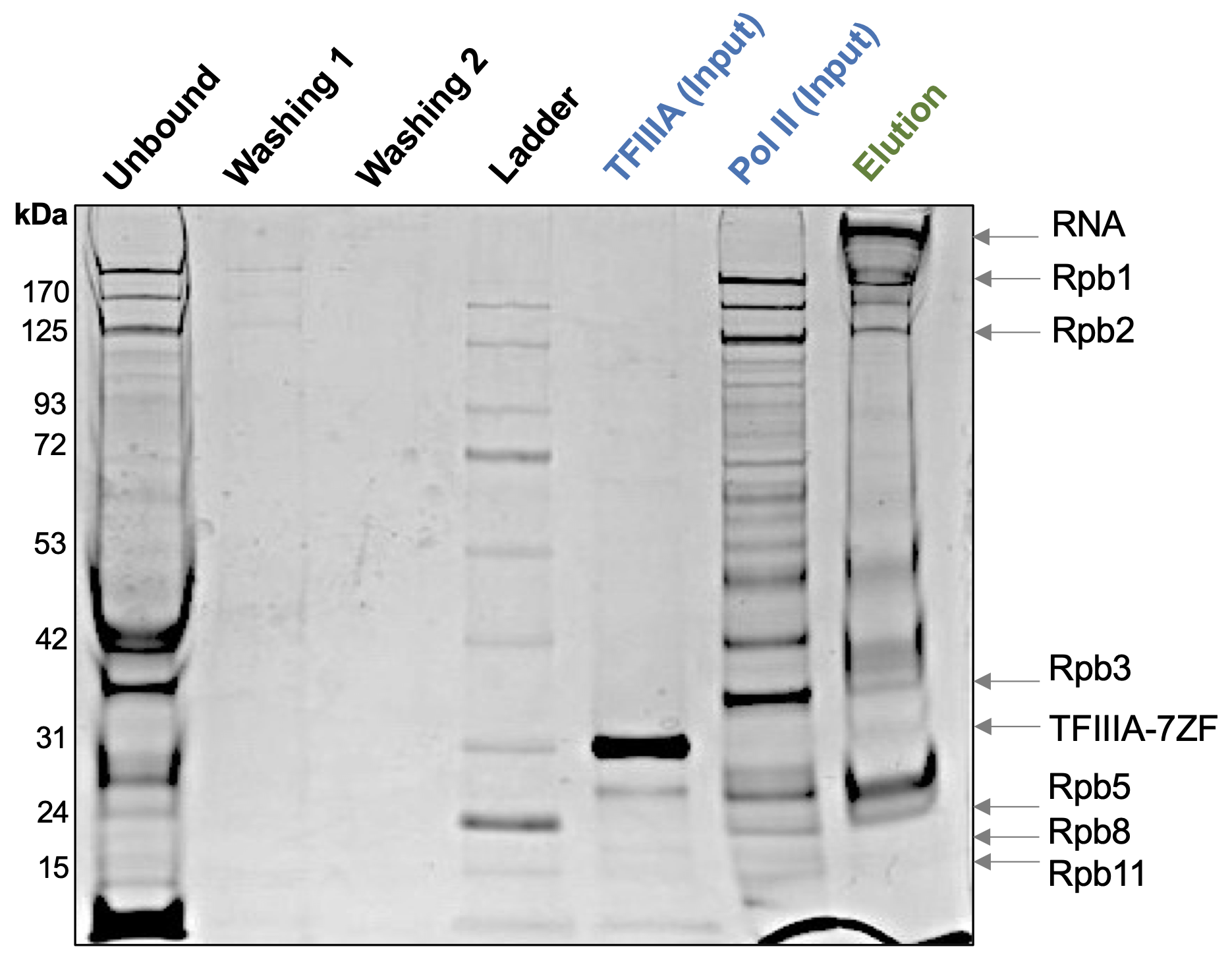

Supplement: S5 Fig — Pol II subunits are labeled based on the predicted molecular weight. (TIF) [file ppat.1010850.s007.tif]
