# Supplementary material for: A remodeled RNA polymerase II complex catalyzing viroid RNA-templated transcription
Source: PLoS Pathog. 2022 Sep 19;18(9):e1010850. doi: 10.1371/journal.ppat.1010850 (PMC9521916; doi:10.1371/journal.ppat.1010850)
Supplement: Attachment — Submitted filename: Response letter.pdf [file ppat.1010850.s015.pdf]

Two out of three reviewers appreciate this work represents an important advance in viroid replication. I personally agree with this appreciation. My suggestion is that you try to clarify the points raised by the reviewers and that, in the Discussion section, comment on future directions to bring this research line to a more in vivo context, as one of the reviewer is demanding.
Response: Thank you. We are grateful for the constructive comments from the editor and the reviewers that help us to improve this manuscript. Please find our specific response below.

Reviewer Comments (if any, and for reference)
Reviewer's Responses to Questions
Part I - Summary
Please use this section to discuss strengths/weaknesses of study, novelty/significance, general execution and scholarship.

Reviewer #1: In this work submitted by Dissanayaka Mudiyanselage et al, is showed (by using an "in vitro" system) that Pol II is able to accept (-)PSTVd RNA template to generate plus-strand vdRNAs. Furthermore, the components of this "in vitro" transcriptionally active complex were analyzed by mass spectrometry to identify their potential components.
The manuscript is well organized and the experimental design is adequate, however the obtained data arise from an artificial system, and the authors cannot determine that similar complex could also exist during viroid infection.
Response: Thank you. We agree with the reviewer that reconstituted in vitro systems (IVT) have certain limitations. We have significantly revised the discussion to clarify this point.

Nevertheless, IVT systems provide a powerful model for screening and characterizing transcription and replication factors, especially for the complicated system that cannot be easily tested in vivo. Here, the gained knowledge provides new insight into PSTVd replication and can guide future studies.

Reviewer #2: This is a well written manuscript that provides relevant evidence on the involvement of RNA polymerase II (Pol II) the in the synthesis of the plus polarity strand of potato spindle tuber viroid (PSTVd), the type member of the viroid family Pospiviroidae, thus addressing and clarifying a still controversial issue regarding the replication of this nuclear viroid through the asymmetric pathway of the rolling circle mechanism. Moreover, the authors showed that the Pol II complex on the viroid RNA template is remodeled with respect to that acting on DNA templates, with several transcription factors reported in the latter lacking in the former complex where a major role to increase the transcription efficiency of the RNA template is played by the transcription factor TFIIIA-7ZF. This manuscript provides an experimental system to further dissect the transcription also of other viroids and viroid-like RNAs, thus being of general interest for the study of these infectious agents and for further dissect the composition of RNA II transcription complexes using the RNA as a template.
Experiments are carefully performed with appropriate replicates and controls
The manuscript deserves publication.

Response: Thank you.

Reviewer #3: This is a well executed study and well written manuscript.
The authors used their IVT system followed by nano-liquid chromatography, and provided evidence that a modified-simplified Pol II complex, aided by TFIIIA-7ZF, can utilize as template (-) PSTVd to produce (+) oligomers.
The study also identified the critical zinc finger domains of the TFIIIA-7ZF for pol II RNA template binding.
This study contributes novel and important information. It performed in vitro experiments with systems that allowed a closer representation of cellular activities (e.g., size of viroid template and product) and provide insights on the adaptation of the DNA dependent RNA pol II to accept as a template (-) sense RNA. The results of the study are also in agreement with the hallmark of the error prone RNA replication as well as the evolutionary movement from the RNA to the DNA world and from prokaryotic to eukaryotic organisms since it identified that (-) sense viroid RNA is utilizing a simplified pol II, lacking a substantial number of sub-units including the ones required for proof reading activity, as well as the absence of several transcription factors that are typically present in eukaryotic cells when DNA is the template for pol II.

In the cases where the study did not produce clear evidence the authors identified shortfalls of the experimental tools and identified the needs for further work.
Response: Thank you.

Part II – Major Issues: Key Experiments Required for Acceptance
Please use this section to detail the key new experiments or modifications of existing experiments that should be absolutely required to validate study conclusions.

Generally, there should be no more than 3 such required experiments or major modifications for a "Major Revision" recommendation. If more than 3 experiments are necessary to validate the study conclusions, then you are encouraged to recommend "Reject".

Reviewer #1: Additionally, some additional controls may contribute to elucidate the transcriptional efficiency of the complex Pol II/PSTVd. For example the use of canonical PSTVd(+) template as comparative transcription control, could be a good parameter to analyze if the activity of this (PSTVd(-)/Pol II) transcriptional complex is comparable or only a residual effect.
Response: Thank you. We think that the observation of the strong aiding effect of TFIIIA-7ZF for enhancing Pol II activity (Figure 1a) is against the possibility of the residual effect. We agree with the reviewer that this comparison is meaningful and desirable. The optimized stoichiometric ratios among Pol II, TFIIIA-7ZF, and RNA templates were reported in Ref. 24 (for circular (+) template) and in this manuscript (for (-) dimer template). Since the optimized concentrations of each component and stoichiometric ratios are different, it is difficult to have a direct comparison. This is now discussed in the fifth paragraph in the revised Discussion.

Due to technical constrain, we cannot use any labeled linear (+) PSTVd template to perform this assay. This is because the Pol II/TFIIIA-7ZF complex has no activity on linear (+) PSTVd template (reported in Ref. 24). It is also difficult to use circular (+) PSTVd template for this assay because global labeling of PSTVd RNA will make it inaccessible to the Pol II/TFIIIA-7ZF complex. We now discuss this issue in the fifth paragraph of the revised Discussion.

On the other hand, internals controls with the WT (TFIIIA-7ZF complex) should be included in the mutants-assays in order to provide an internal control that allowed perform a true comparative analysis of the transcriptional activity between WT and mutants. For example according the figures showed, this reviewer consider that the transcriptional activity of the WT complex (Fig 1A), is comparable or minor that the observed with the mutants zf4 and zf7.

Response: Sorry for the confusion. The images for mutants zf4 and zf7 in Figure 3 were exposed longer as compared with the WT due to the weak signals. As evidence, the “Pol II only” lane in the images for mutants zf4 and zf7 had similar signal strength as compared with those with mutant proteins. For signal quantification, we always used enhanced signals aided by the supplement of TFIIIA-7ZF proteins compared with the corresponding signals with only Pol II in the same blot. Therefore, mutants zf4 and zf7 did not enhance Pol II activity in contrast to the strong activity of WT TFIIIA-7ZF in enhancing Pol II transcription. Statistical analysis also supports our conclusion. We now explain the quantification method in the second paragraph of the Result section.

We agree with the reviewer that ideally a WT positive control should be included for all assays. Even though a WT treatment was not included, we are confident about our data. Because there are at least three biological replicates for all mutants and the WT sample that were performed during a similar time period. Data quantification and statistical analyses provide additional support for our conclusion.

Finally, the figures are poorly explicative, making hard the interpretation of the results. For example, internals size controls are missing, the authors claim about the longitude of the obtained transcripts, but size references (transcripts obtained by conventional transcription from dimeric clones) are no provided.

Response: Sorry for the confusion. We could observe the extremely weak signal of the template that only showed up after over-exposure when we detected the product, which is likely caused by the weak non-specific cross-reaction of riboprobes (shown in the new S4 Figure). We relied on those template signals to estimate the size of our products. The product signals were often retarded in gel running caused by high-salt/high-loading-volume condition. This is explained in the legend of S4 Figure. Since Pol II alone could produce a product similar to the template size in all replicates for all TFIIIZ-7ZF mutants (21 replicates), the product size generated by Pol II without WT TFIIIA in Figure 1a should be similar to the template size. In addition, when we were optimizing the condition, we had one trial with WT TFIIIA-7ZF that included size markers (see Image 1). We are confident that our (+) PSTVd product is similar to (-) dimer in length. Just to be cautious, we revised the manuscript to avoid using a specific length to describe the product.

We did not include Image 1 in the manuscript since the main information is already in S4 Figure. However, we will be happy to include it if the reviewers and the editors feel necessary.

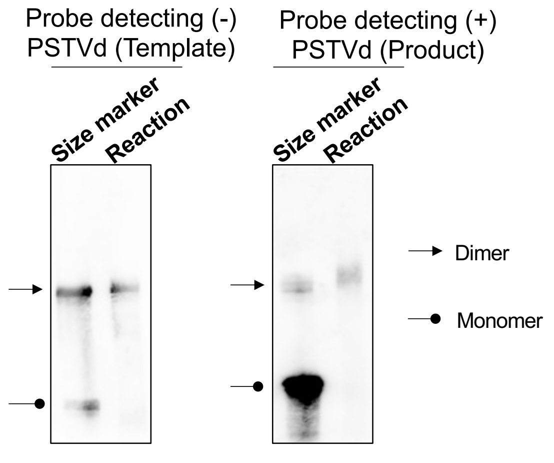

Reaction: 5.4 nM Pol II, 30 nM TFIIIA-7ZF and 7.8 nM RNA.

Image 1

Reviewer #2: I think that the point reported below should be addressed by the authors to increase the clarity and the completeness of the manuscript:
My major concern regards the dimeric RNA template used in the reported experiments. It has been described in details in a previous publication. However, in the context of this manuscript is it is very important to provide a clear description of such a template and possibly address more in details (maybe in the discussion) several issues that likely will be further investigated in the future.
Response: Thanks for pointing it out. We now include a new S2 Figure to illustrate the construct for PSTVd dimer and the extra non-viroid sequences in our RNA template. We also discuss the presence of the non-viroid sequences in the sixth paragraph of the revised Discussion.

The authors should clarify somewhere in the main text whether such a template includes non-viroid sequences at its terminal ends. Which are the initial and terminal positions of the used template should also be clarified. Do the authors expect the same transcription efficiency (or hte same size of products) using dimeric templates starting at different positions? Related to this is the question of whether a promoter does exist on this template and whether the authors retain that their system may help to clarify this point.
Response: Thanks. We have explained the sequences of the RNA template in the first paragraph of the Result section. We discuss the potential RNA promoter and possible future studies in the sixth paragraph of the Discussion. Since we focus on introducing the IVT system, the composition of the remodeled Pol II, and the functional domains in TFIIIA-7ZF, the test for transcription efficiency using different templates is discussed for future investigations.

Another point regards the size of the generated RNA (more than 700 nt).
A first concern is that in the reported figures it is not possible to appreciate the size of the transcript f. A figure representing the complete gel could be provided as supplementary material to appreciate the marker and the absence of additional (maybe shorter?) transcription products.
Response: Thank you. As explained in detail to the similar question raised by reviewer 1, we could observe the extremely weak signal of template that only showed up after over-exposure when we detected the product, which is likely caused by the weak non-specific cross-reaction of riboprobes (shown in the new S4 Figure). We relied on those template signals to estimate the size of our products. In addition, Image 1 also shows

that the product from the activity of the Pol II/TFIIIA-7ZF complex is similar in size to PSTVd dimer.

Another issue, always related to this point, is the conclusion by the authors (Line 267) that in their experimental system the transcription “is terminated by template run-off”. It does not seem that experimental data are provided to support this conclusion. This point should be better clarified, highlighting the data supporting such a conclusion.
Response: Thank you. We now revised the text to “However, the mechanism underlying transcription termination on viroid RNA template remains unknown. Thus, it is unclear whether our reconstituted IVT system reflects the transcription termination mechanism occurring in plants. Future investigations are needed to dissect the machinery and mechanism involved in viroid RNA-templated transcription”.

Reviewer #3: I do not see any major issues with the study. The experimental systems are well established and this study is building and expanding on previous studies with proven results.
Response: Thank you.

Part III – Minor Issues: Editorial and Data Presentation Modifications
Please use this section for editorial suggestions as well as relatively minor modifications of existing data that would enhance clarity.

Reviewer #1: (No Response)

Reviewer #2: L 25:I would avoid the use of mysterious here. Viroids are quite well characterized infectious agents as stated below.
Response: Thanks. We use “fascinating” replacing “mysterious”.

L29-30: Maybe clarify here that the involvement of Pol II in the synthesis of minus strand has been already addressed and confirmed.
Response: Thanks. We add “It is well-known that” to the sentence mentioned.

L120: How the size of the transcribed sequences were confirmed? No marker in the figure is shown (the full gel could be provided as supplementary figure. In addition, the fact that the identity was confirmed by Northern blot hybridization is reported in the legend of the figure, I suggest to clarify this also in the main text.
Response: Thanks. The suggested supplementary figure is included as the new S4 Figure. We added the size information in the legend of the S4 Figure. We mentioned that the product identity was confirmed by RNA gel blots in the second paragraph of the Result section.

Reviewer #3: 1. This study is building on previous studies of the same group. For a first time reader however, that has not digested the previous studies (e.g., 23, 24) it could be challenging to follow the experiments sequence.
For example, was Pol II purified from wheat once and a portion was studied for its "WT"

properties/complex structure and another portion was used in the IVT system with (-) PSTVd template to study the remodeled Pol II complex structure?

OR

Is the IVT system is more of a "kit/off the self" kind of system?
In addition, while Pol II is generated in planta other elements of the study (i.e., transcription factors) are generated in vitro/bacterial systems. This also could be a source of confusion.
Response: Thanks. The Pol II was purified from wheat germ using a protocol we optimized and published recently (Ref. 24). After purification, we used aliquots for each assay. Based on our experience, the activity will last about 3 months. So, we made Pol II prep every three months. Each prep was confirmed and quantified by using western blot to detect the largest subunit of Pol II. TFIIIA-7ZF proteins were purified from the bacterial expression system as previously reported.

The remodeled Pol II was purified using a biotin-labeled RNA template. As described in the third paragraph in the Results and Figure 1b, the partially purified Pol II (from wheat germ) was added to a biotin-labeled RNA template, washed, and eluted for nLC-MS/MS analysis. While both partially purified Pol II and the remodeled pol II have activity, the remodeled Pol II has a minimal set of factors for RNA-templated transcription.

This is a classic reconstituted in vitro assay (without an off-the-shelf kit). We include a new S1 Figure to illustrate the experimental flow.

Maybe a small schematic/flowchart, perhaps as a supplemental figure, of the experimental approach and how all the different elements, come together and how they are analyzed, can improve the reader's experience.
Response: Thanks for the suggestion. We have included a new S1 Figure to illustrate the experimental flow.

2. Please check #51 reference. Is it the correct/best one for the PSTVd dimer construction?
Response: Thanks. The original construct was made for the project that has been published as the #51 reference (in original submission). However, as the reviewer pointed out, the publication did not include any data based on the construct. So, we included a new S2 Figure to illustrate the construct. We also stated in the revised text (Methods) that this construct was inherited from late professor Biao Ding.
